# Determination of lymph node metastasis using quantitative ultrasound elastography of papillary thyroid carcinoma nodule: a systematic review and meta-analysis

**DOI:** 10.1186/s12880-025-01858-z

**Published:** 2025-08-21

**Authors:** Alisa Mohebbi, Saeed Mohammadzadeh, Mohammad Ghaffari, Afshin Mohammadi, Nathalie J. Bureau, Ali Abbasian Ardakani

**Affiliations:** 1 Department of Radiology, Tehran University of Medical Sciences, Imam Khomeini Hospital, Tehran, Iran; 2 School of Medicine, Tehran University of Medical Sciences, Tehran, Iran; 3 Department of Radiology, Faculty of Medicine, Urmia University of Medical Science, Urmia, Iran; 4 Research Center, Centre Hospitalier de l’Université de Montréal CA, Montreal, QC Canada; 5 Department of Radiology Technology, School of Allied Medical Sciences, Shahid Beheshti University of Medical Sciences, Tehran, Iran

**Keywords:** Ultrasound elastography (USE), Papillary thyroid carcinoma (PTC), Malignant nodules, Diagnostic test accuracy (DTA)

## Abstract

**Background and purpose:**

papillary thyroid carcinoma (PTC) as the most common thyroid tumor, tends to invade adjacent organs, especially lymphatic system. This study aimed to evaluate the discrimination performance of ultrasound elastography (USE) in assessing PTC nodule for determination of cervical lymph node metastasis (CLNM).

**Methods:**

The protocol was pre-registered at (https://osf.io/r5tc8). Using PubMed, Web of Science, Embase, and Cochrane Library, studies published up to March 10, 2025, were identified. Data extraction was conducted independently, and a random-effects bivariate model was applied to estimate pooled differentiation accuracy estimates.

**Results:**

Twenty-one studies were included involving 7559 patients; 2790 (36%) were positive for CLNM, while 4769 (63%) were negative. The pooled E_mean_ values for positive and negative CLNM were 51.2k Pa (95% CI: 42.6 to 59.7) and 44.8 kPa (95% CI: 35.2 to 54.4), respectively. It represents an absolute increase of 6.14 kPa (95% CI: 2.70 to 9.59) in the metastatic group compared to the benign group. Additionally, the pooled E_max_ value for positive and negative CLNM were 87.9 kPa (95% CI: 49.5 to 126.4) and 68.7 kPa (95% CI: 44.2 to 93.1), respectively. This corresponds to an absolute increase of + 19.57 kPa (95% CI: 2.96 to 36.18) in the metastatic group, representing a more dramatic elevation compared to E_mean_ values. The thyroid nodule E_max_ and E_mean_ were significantly higher for positive CLNM of + 27.5% (95% CI: 10.5–44.5%) and + 12.9% (95% CI: 5.1–20.7%) respectively. Combining USE with conventional ultrasound improved differentiation accuracy, achieving a sensitivity of 80% (95% CI: 62–90%), specificity of 79% (95% CI: 70–85%), and an AUC of 0.85 (95% CI: 0.81 to 0.88).

**Conclusion:**

USE parameters demonstrated potential as a discrimination tool for the preoperative assessment of CLNM, particularly when combined with conventional ultrasound, which enhances its performance.

**Clinical trial number:**

N/A.

**Supplementary Information:**

The online version contains supplementary material available at 10.1186/s12880-025-01858-z.

## Introduction

Papillary thyroid carcinoma (PTC) is the most common type of thyroid cancer, accounting for approximately 80% of diagnosed thyroid malignancies [1]. In 2020, the global age-standardized incidence rate of PTC was 10.1 for women and 3.1 for men per 100,000, representing about 2.2% of all new cancer cases diagnosed annually in the United States [1]. Although PTC can affect children and the elderly, it is more prevalent in the 45–64 age range. The prognosis and 5-year survival rates are favorable, yielding an overall 5-year survival of 97.5% and 10-year survival of 93% [2, 3].

Clinically, PTC is usually presented as an irregular, solid, slow-growing mass, often with microcalcifications and rarely with cystic features [4]. It is often diagnosed as an incidental finding in imaging performed for unrelated reasons. However, symptomatic patients usually present with a thyroid nodule, pain from internal hemorrhage within the nodule, or enlarged cervical lymph nodes [5]. The diagnostic approach typically begins with an initial ultrasonography assessment, sometimes combined with fine needle aspiration (FNA) and histopathological examination as the gold standard for definitive diagnosis [6].

PTC tends to invade adjacent organs, especially the lymphatic system. Different lymphatic networks, including the central, lateral, and collateral lymphatic networks, are subjected to its invasions. However, the most frequent site of metastasis is the central lymph nodes, occurring in 30–90% of cases [7]. This underscores the critical role of accurate cervical lymph node metastasis (CLNM) detection in guiding surgical strategies and improving prognostic outcomes.

Early diagnosis and detection of central lymphatic metastasis are crucial, as prognosis declines significantly with disease progression and metastasis. Consequently, there has been a growing emphasis on developing diagnostic methods that are accurate, reliable, inexpensive, accessible, and minimally invasive. Among these, ultrasound elastography (USE) has emerged as a promising technology in the medical field. USE provides morphological information, elasticity values, and mechanical properties of connective tissue, enabling the differentiation of masses [8]. The unique pathological features of PTC—characterized by loose connective tissue stalks covered by one or more malignant cells—result in distinct elastic properties compared to normal thyroid tissue and benign lesions. These differences make USE a plausible detection tool for more accurately identifying involved central lymph nodes [9]. In this study, we systematically evaluate the discrimination accuracy of the USE examination of PTC nodules among the reviewed studies, aiming to clarify its potential role in improving early and accurate detection of CLNM.

## Materials and methods

We conducted our methodological strategy following the comprehensive guidelines outlined in the Cochrane Handbook for Systematic Reviews of Diagnostic Test Accuracy. This handbook provides detailed methodologies and the latest practices for conducting systematic reviews that evaluate the accuracy of diagnostic tests. Our study also complies with the Preferred Reporting Items for Systematic Review and Meta-analysis of Diagnostic Test Accuracy (PRISMA-DTA) guidelines to ensure standardized reporting of our findings. Additionally, we utilized the PRISMA-Search (PRISMA-S) guidelines to standardize our search approach, facilitating a systematic and thorough search for relevant studies. To enhance transparency and reproducibility, the study protocol was also pre-registered on the Open Science Framework (OSF) at the URL *“*https://osf.io/r5tc8” before initiating the research (see Appendix A). This pre-registration step is crucial as it helps prevent bias by outlining methods and objectives in advance, ensuring that the research is conducted transparently and considers potential issues.

### Search strategy

Two reviewers independently developed search strategies using EMTREE and MeSH keywords for exploration. After creating their individual syntaxes, the strategies were reviewed and integrated to form a cohesive and robust search strategy. A discussion with the third reviewer resolved any disagreements. A comprehensive search was conducted through online databases, including PubMed, Web of Science, Embase, and Cochrane Library, on March 10, 2025. The keywords in the search included “papillary thyroid carcinoma,” “lymph node,” and “elastography.” The full search strategy is provided in Appendix B. No language restrictions were applied. Additionally, the reference lists of relevant papers were examined to ensure a thorough assessment and identify any possibly missed publications. All the data were ultimately imported into EndNote software for manual duplicate study elimination.

### Eligibility criteria

Observational (cross-sectional, case-control, cohort) and experimental (randomized and non-randomized trial) study designs were evaluated and included in this systematic review. Other studies, such as case reports/series, editorials, comments, correspondence, guidelines, meta-analyses, systematic and narrative reviews, and grey literature published outside peer-reviewed journals, were excluded. Animal experiments or in vitro studies were also excluded. Studies assessing the accuracy of USE thyroid nodule detection for identifying CLNMs using quantitative USE parameters (e.g., E_mean_, E_max_) were eligible for our review. Histopathological findings of lymph nodes had to be used as the gold standard; otherwise, the study was excluded. Additionally, studies that only reported the qualitative (i.e., visual) role of USE were also excluded. Papers investigating head and neck malignancies other than PTC were excluded. PTC cases needed confirmation with thyroid nodule histopathology. The authors’ names in the listed studies were also cross-checked to avoid including duplicate reports. If multiple studies utilized the same or a subset of patients, the paper with the more complete and larger sample was retained.

### Risk of bias assessment

To ensure the accuracy and reliability of our synthesized results, each paper included in our study was independently evaluated for bias risk by two reviewers using the Quality Assessment of Diagnostic Accuracy Studies-2 (QUADAS-2) tool. This tool is widely recognized for its effectiveness in assessing the risk of bias across four critical domains:

- Patient selection: This domain evaluates whether the study population was appropriately selected and representative of the target population.
- Index test: This domain assesses whether USE was performed and interpreted correctly.
- Reference standard: This domain examines whether the reference standard used to confirm the diagnosis was appropriate and applied consistently to participants.
- Flow and timing: This domain assesses whether the interval between the index test and reference standard was appropriate, ensuring that the results of the index test were not affected by changes in the condition being detected over time. We determined a maximum ≤ 1-month cutoff between index test and reference standard to assess the flow and timing bias.

### Data extraction

Initially, the reviewers created an Excel data extraction form that included essential information for the systematic review, ensuring clarity and consistency. Basic study details (authors, publication year, originating country, study design, USE method, number of radiologists, and their experience) along with population parameters (age, thyroid nodule size, sample size, number of patients with positive and negative CLNMs) were extracted and imported into the predefined form. The E_mean_ and E_max_ parameters of thyroid nodules were also evaluated. Definitions of cases were as follows:

- True positive cases: PTC patients with positive CLNM as determined by USE and positive CLNM as confirmed by histopathology.
- True negative cases: Negative CLNM as determined by USE and negative CLNM as confirmed by histopathology.
- False positive cases: PTC patients with positive CLNM as determined by USE and negative CLNM as confirmed by histopathology.
- False negative cases: PTC patients with negative CLNM as determined by USE and positive CLNM as confirmed by histopathology.

The Digitizer web plot was also used to extract individual participant data from scatter plots in the included articles if necessary.

### Data synthesis

In our analysis, we faced a challenge where only one of the two formats—kPa (kilopascals) and m/s (meters per second)—was reported in each of the included studies. To address this and standardize the results, we utilized the formula E = 3ρV² to convert velocity (m/s) to stiffness (kPa). In this formula, ρ represents the density of the lymph node, which is approximately 1.03 g/cm³, E represents stiffness in kPa, and V represents velocity in m/s. We focused on evaluating the main elastography parameters, including E_mean_ and E_max_, which measure the average and maximum stiffness of the thyroid nodule, respectively. To compare these parameters between groups with positive and negative CLNM, we calculated the mean difference (MD), standardized mean difference (SMD), and percentage difference. This allowed us to determine whether these parameters were higher or lower in the presence of metastasis. Subgroup or meta-regression analyses were conducted based on several factors, including the risk of bias as assessed by the QUADAS-2 tool, nodule size, age, sex, USE method, and study design (i.e., retrospective vs. prospective). These would help determine how different factors might influence the discrimination accuracy of elastography.

We employed software tools for statistical analysis, including STATA version 17.0, MedCalc version 20.0, and Psychometrica. A bivariate random-effects pooling model combined the results from various studies, allowing us to account for variability between studies. Sensitivity analyses were performed to assess the robustness of our findings, and publication bias tests were conducted to evaluate whether there was a tendency for studies with specific results to be more likely to be published. The trim & fill method was used to correct for publication bias if detected.

We used standard interpretation zones reported in the literature to interpret the magnitude of our observed effects. We calculated discrimination values such as the area under the curve (AUC), sensitivity, specificity, and diagnostic odds ratio (DOR). Receiver operating characteristic (ROC) curves were plotted to assess the visual classification performance of elastography. If studies provided 2 × 2 contingency table data for each radiologist, we pooled them into an overall 2 × 2 contingency table before including them in the meta-analysis. This allowed us to synthesize the results from multiple radiologists into a single estimate of discrimination performance. High statistical heterogeneity was defined as an I2 value of 50% or more, indicating substantial variability between studies. A p-value of 0.05 was used as the threshold for determining statistical significance in tests of effect size, specifically for comparing mean differences in elastography parameters (Emean and Emax) between groups with positive and negative cervical lymph node metastasis.

## Results

**Fig. 1:**
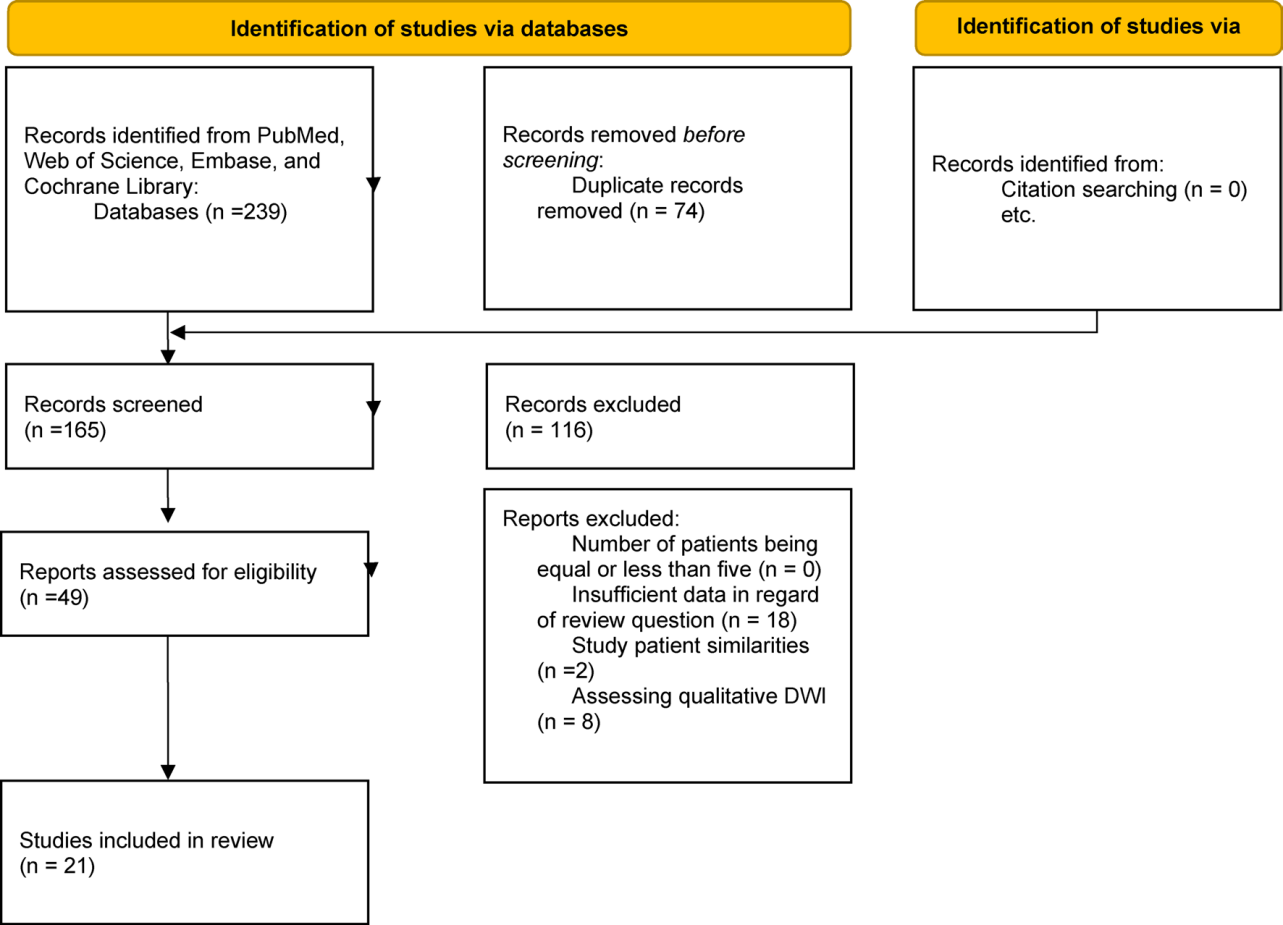
PRISMA flowchart for study eligibility

Search results and study characteristics:

A total of 7559 patients with 7592 thyroid nodules from 21 studies were included in the present study. Among these, 2790 (36.9%) patients had positive CLNM, while 4769 (63.1%) were negative [10–30]. Of the 21 studies in our review, 18 originated from China and 3 from South Korea. Among the included studies, 5 utilized the acoustic radiation force impulse (ARFI) method, 1 employed the real-time elastography (RTE) technique, and 15 implemented the shear wave elastography (SWE) approach. The detailed characteristics of the studies are summarized in Table 1. Also, the PRISMA flow diagram, illustrating the selection process, is provided in Fig. 1.

**Table 1:** Characteristics of included studies

Authors	Year	Type of elastography	Age	Nodule number	Patient number	Country	Study type	Number of patients with negative LNM	Number of patients with positive LNM	Number of radiologists	Experience of radiologists (years)
Xu et al. (2015)	2015	ARFI	44 ± 13 for positive LNM and 50 ± 11 for negative LNM	222	203	China	Retrospective	125	78	5	NR
Xu et al. (2016)	2016	ARFI	48.8 ± 12.1	252	252	China	Prospective	180	72	2	9
Park et al. (2016)	2016	SWE	44 (range 15–74)	363	363	South Korea	Retrospective	233	130	2	10 and 15
Yoon et al. (2017)	2017	RTE	43.7 ± 10.1	79	79	South Korea	Retrospective	65	14	3	7 to 15
Zhang et al. (2020)	2020	SWE	45.24 ± 8.56	553	553	China	Retrospective	452	101	2	7
Xu et al. (2020)	2020	ARFI	50.4 ± 14.0	116	116	China	Prospective	74	42	1	> 10
Guo et al. (2020)	2020	SWE	44.9 ± 9.4	433	433	China	Retrospective	279	154	2	> 5 for one reader
Li et al. (2020)	2020	ARFI	36 (IQR 31–42)	510	510	China	Retrospective	297	213	2 sonographers	> 10
Li et al. (2020)	2020	SWE	47.2 ± 10.2	172	172	China	Retrospective	89	83	2	> 5
Han et al. (2020)	2020	SWE	48.9 ± 11.9	111	111	South Korea	Retrospective	67	44	3	4, 7, 11
Huang et al. (2022)	2022	SWE	42 (IQR 31.25–53)	387	387	China	Retrospective	276	111	NR	NR
Zhang et al. (2023)	2023	ARFI	49.53 ± 13.68 for positive LNM and 51.57 ± 11.57 for negative LNM	101	101	China	Retrospective	61	40	2	> 5
Wang et al. (2022)	2022	SWE	139 of 206 patients were > 45	206	206	China	Prospective	123	83	1	> 5
Xue et al. (2023)	2023	SWE	44.46 ± 9.98 for training set and 43.15 ± 10.70 for validation set of positive LNM and 43.71 ± 9.80 for training set and 44.53 ± 11.98 for validation set for negative LNM	129	129	China	Retrospective	68	61	2 sonographers	> 10
Wan et al. (2023)	2023	SWE	42.2 (range 19–72)	148	142	China	Retrospective	73	75	More than 1	> 10
Liu et al. (2023)	2023	SWE	45 (range 22–75)	541	541	China	Retrospective	343	198	2 sonographers	> 10
Liu et al. (2023)	2023	SWE	43.09 ± 0.54	487	487	China	Retrospective	295	192	1	10
Xu et al. (2023)	2023	SWE	45.60 ± 11.97	50	50	China	Prospective	34	16	2 sonographers	> 5
Xue et al. (2024)	2024	SWE	44.3 ± 10.1	114	106	China	Retrospective	58	48	2 Physicians	> 10
Hu et al. (2024)	2024	SWE	46 (IQR 21–74)	2492	2492	China	Retrospective	1516	976	NR	NR
Jia et al. (2024)	2024	SWE	47.34 ± 9.16 for positive LNM and 46.83 ± 8.54 for negative LNM	126	126	China	Retrospective	67	59	NR	NR

### Risk of bias assessment

**Fig. 2:**
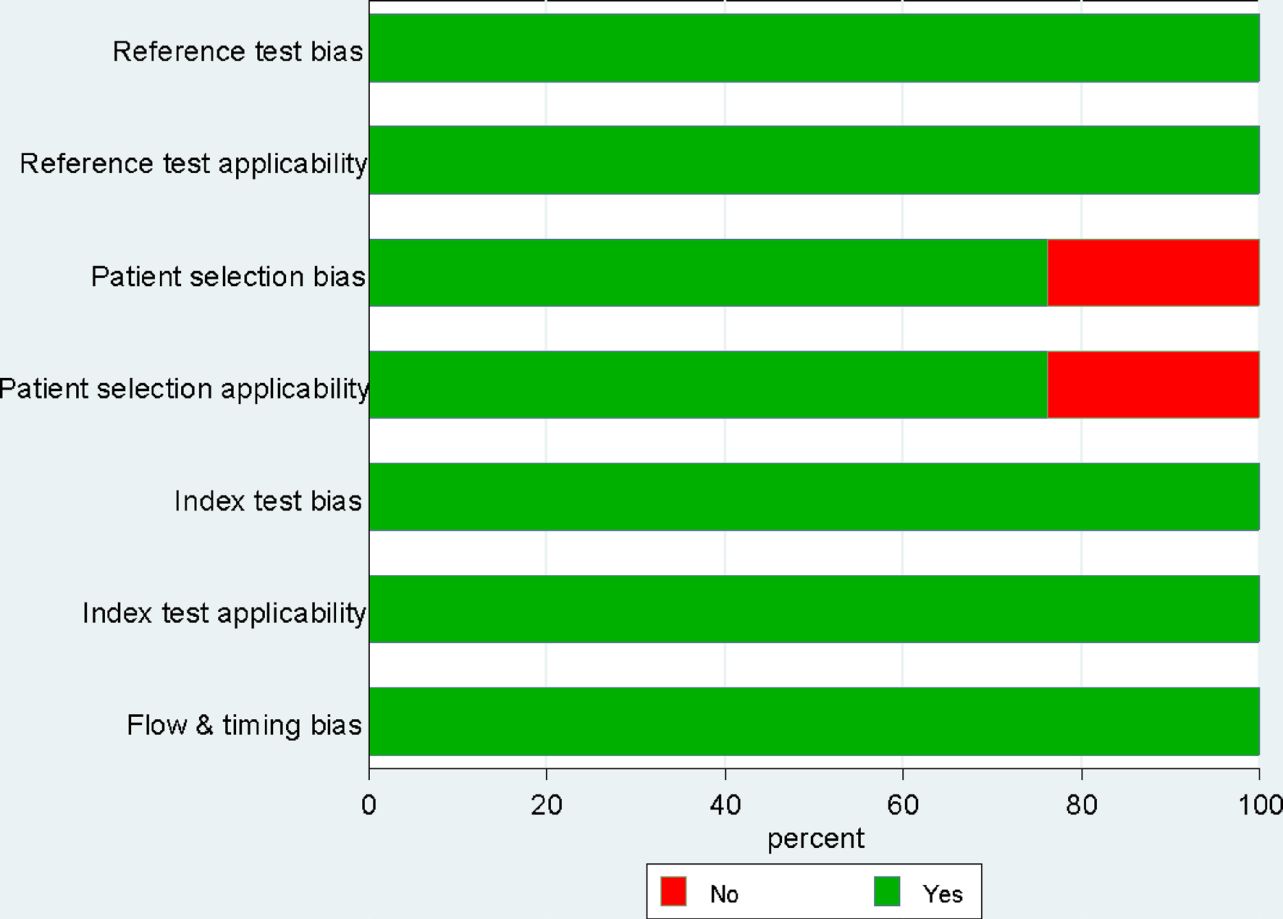
Risk of bias assessment based on QUADAS-2

In regards to QUADAS-2, as shown in Fig. 2, the primary concern identified was the patient selection domain. This bias was primarily attributed to inappropriate exclusions, such as excluding patients with macrocalcifications, limiting the study to patients with the BRAF gene mutation, excluding patients with previous neck dissections, or only including cases with unilateral lymph node involvement. These exclusions could introduce bias by not fully representing the population relevant to the review question.

### E_mean_ findings

**Fig. 3:**
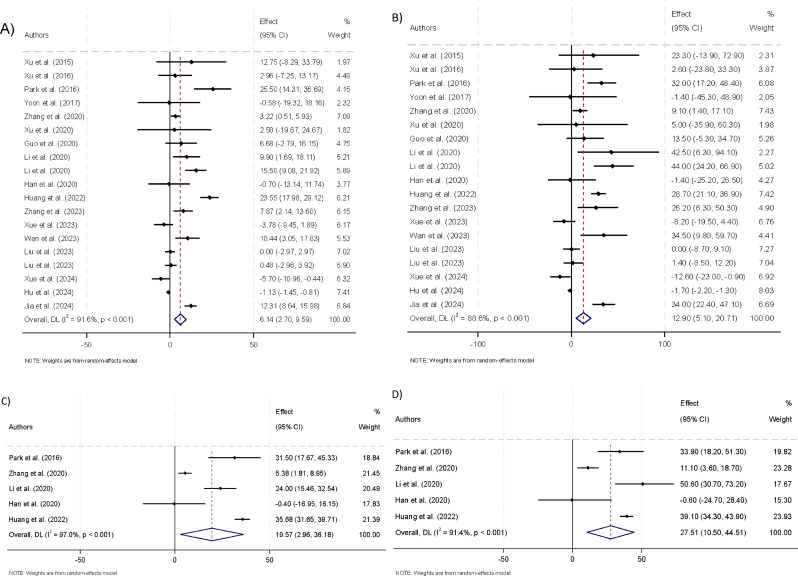
**A**) The mean difference of thyroid nodules E_mean_ with positive and negative lymph node metastasis (CLNM). **B**) The percentage difference of thyroid nodules E_mean_ with positive and negative CLNM. **C**) The mean difference of thyroid nodules E_max_ with positive and negative CLNM. **D**) The percentage difference of thyroid nodules E_max_ with positive and negative CLNM

Of the 21 studies included in the overall meta-analysis, 19 studies provided suitable data for this specific analysis. Across 19 studies included [10–21, 23–26, 28–30], the pooled E_mean_ for thyroid nodules without CLNM in PTC was 44.88 kPa (95% CI: 35.28 to 54.49). In contrast, thyroid nodules with metastasis exhibited a higher stiffness of 51.22 kPa (95% CI: 42.66 to 59.78). The mean difference between the positive and negative CLNM groups was + 6.14 kPa (95% CI: 2.70 to 9.59, p-value < 0.001). The standardized mean difference was + 0.22 (95% CI: 0.04 to 0.40, *P* = 0.019), indicating a small but significant effect size. Additionally, the E_mean_ of the positive CLNM group was + 12.90% (95% CI: 5.10–20.71%, *P* < 0.001) higher compared to the negative CLNM group, suggesting that positive CLNM nodules were stiffer than their negative counterparts (Fig. 3).

**Fig. 4:**
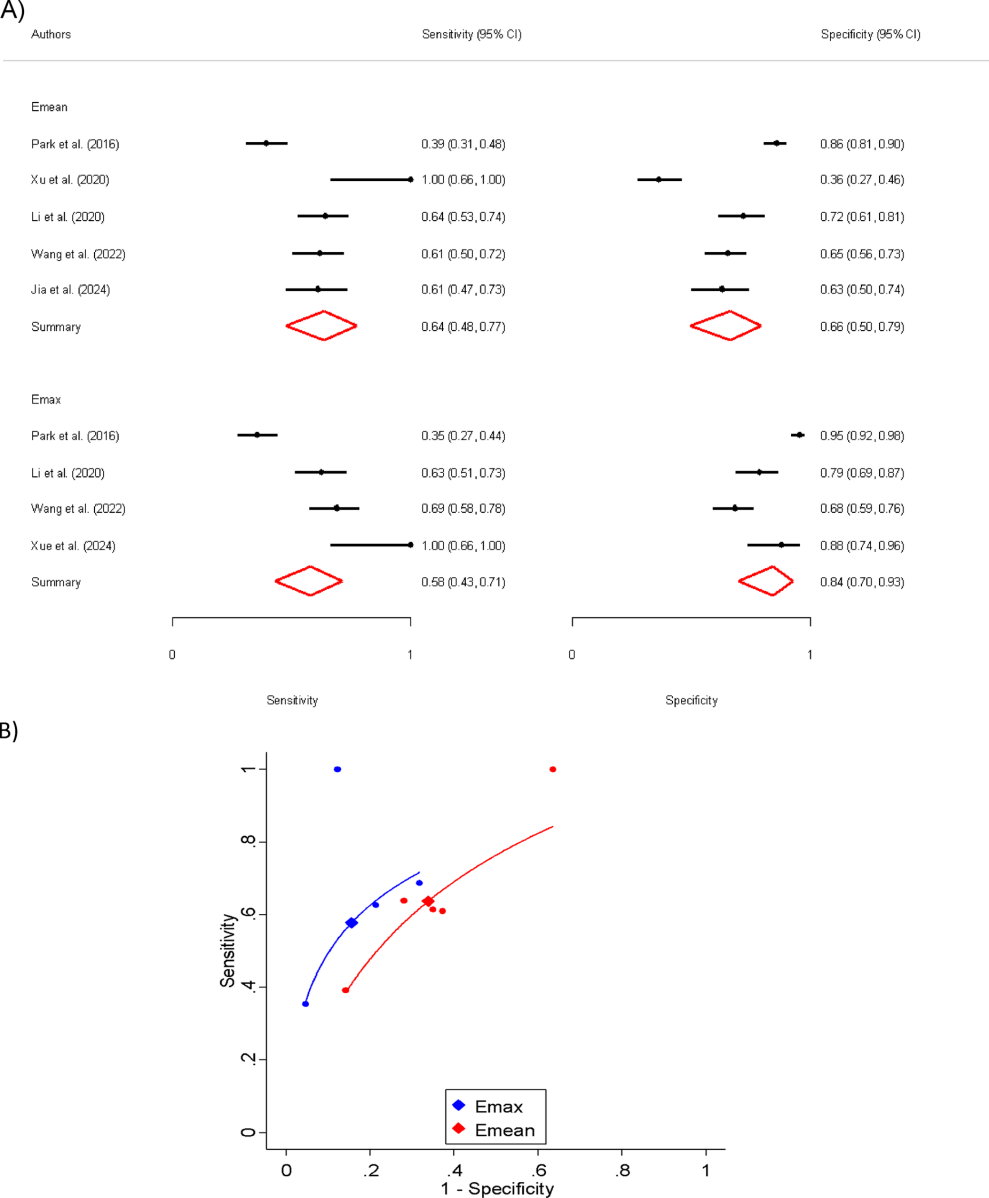
**A**) Sensitivity and specificity for E_mean_ and E_max_ in differentiating positive and negative CLNMs **B**) ROC curve for E_mean_ and E_max_ in differentiating positive and negative CLNMs

The pooled sensitivity and specificity were 64% (95% CI: 48–77%) and 66% (95% CI: 50–79%). The AUC was calculated at 0.69 (95% CI: 0.65 to 0.73, generalized I2 = 0%) (Fig. 4). Sensitivity analysis assessed whether any single study disproportionately influenced the overall results. These analyses revealed that no individual study substantially impacted the USE performance, ensuring the robustness of the findings (Appendix C). Furthermore, tests for publication bias indicated no evidence of bias.

**Fig. 5:**
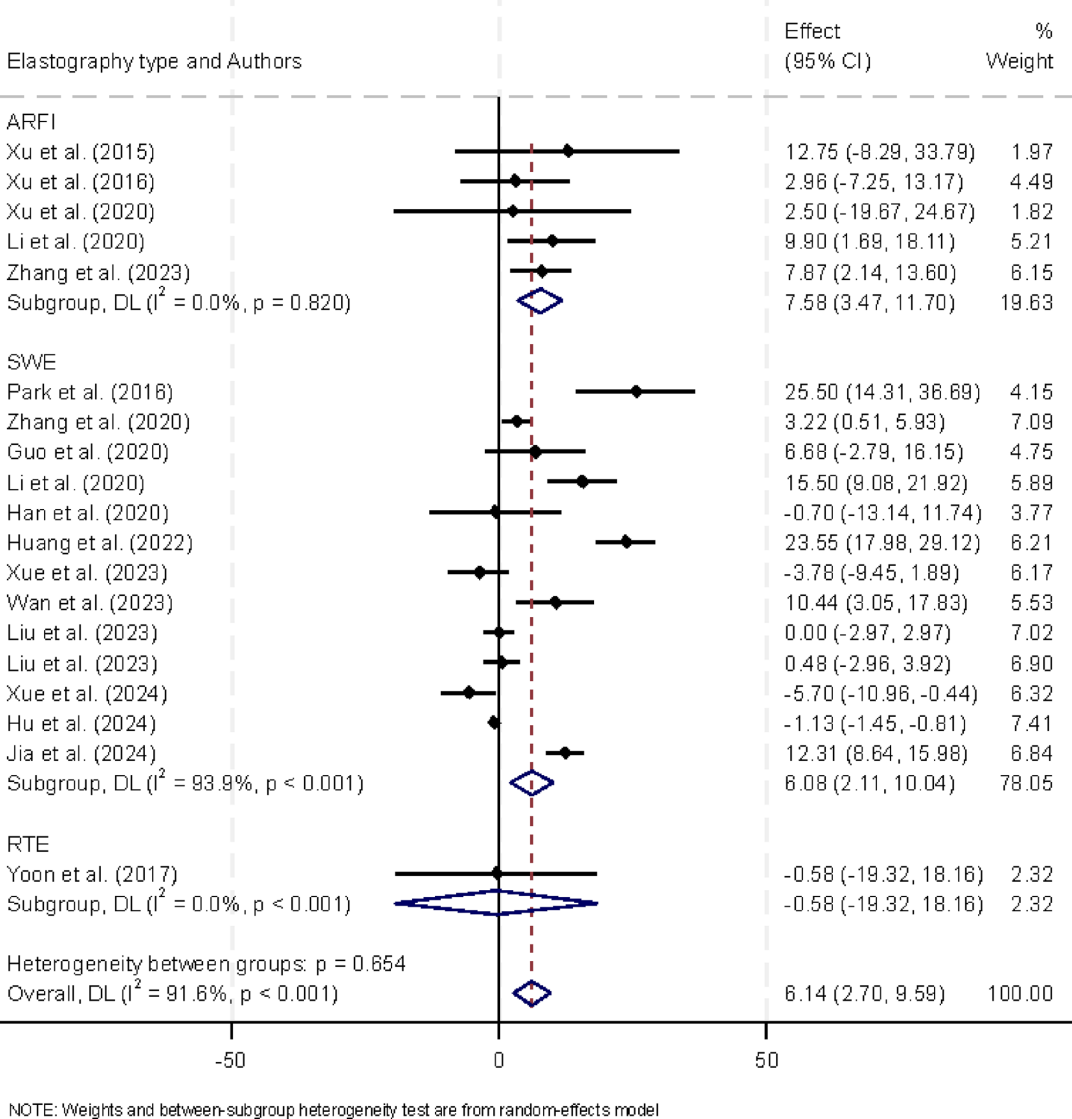
The mean difference of thyroid nodules with positive and negative CLNM based on elastography method

We conducted a subanalysis comparing ARFI, SWE, and RTE to evaluate the performance of various elastography techniques. The results are presented in Fig. 5. This subanalysis showed no substantial difference between ARFI and SWE in distinguishing between positive and negative CLNM. For ARFI, the pooled difference was + 7.58 kPa (95% CI: 3.47 to 11.70, *P* < 0.001), while for SWE, it was + 6.08 kPa (95% CI: 2.11 to 10.04, *P* < 0.001). Additionally, we examined whether study design (retrospective vs. prospective), thyroid nodule size, sex, and age affected the outcomes. However, these factors did not show significant differences in the results, suggesting that the detection performance of elastography for CLNM was consistent across these variables.

### E_max_ findings

In our analysis of Emax values, five studies were assessed to compare the stiffness of thyroid nodules with and without CLNM [12, 14, 18–20]. The pooled Emax for thyroid nodules without CLNM was 68.70 kPa (95% CI: 44.25 to 93.16), while for those with CLNM, it was significantly higher at 87.96 kPa (95% CI: 49.51 to 126.41). The mean difference in E_max_ between positive and negative CLNM groups was + 19.57 kPa (95% CI: 2.96 to 36.18, *P* = 0.021), indicating that nodules with positive CLNM were stiffer than those without metastasis. Furthermore, the standardized mean difference was 0.87 (95% CI: 0.12 to 1.62, *P* = 0.023), which suggests a substantial difference between the two groups. The percentage difference in E_max_ between positive and negative CLNM groups was + 27.51% (95% CI: 10.50–44.51%, *P* = 0.002), further supporting the finding that positive CLNM nodules were stiffer (Fig. 3).

The pooled sensitivity and specificity were 58% (95% CI: 43–71%) and 84% (95% CI: 70–93%). The AUC was calculated at 0.76 (95% CI: 0.72 to 0.79) (Fig. 4). Notably, the differences observed for E_max_ were substantially higher than those for E_mean_. This suggests that E_max_ may be a better USE indicator of CLNM than E_mean_ with a statistically significant p-value (0.009).

### Added value of USE into the conventional US

The integration of USE into conventional US was evaluated in six studies [12, 18, 21, 23, 24, 30]. The pooled results indicated a sensitivity of 80% (95% CI: 62–90%) and a specificity of 79% (95% CI: 70–85%). The AUC was 0.85 (95% CI: 0.81 to 0.88), suggesting that combining USE with conventional US improves classification accuracy compared to the previous values for E_mean_ and E_max_ (generalized I2 = 0.09%) (Appendix D).

## Discussion

In the present study, we aimed to investigate the diagnostic accuracy of USE for detecting central lymph node involvement in PTC. Two measures, E_mean_ (average elasticity measurement) and E_max_ (maximum elasticity measurement), were extracted and analyzed. Our findings indicate that E_max_ demonstrated superior diagnostic performance compared to E_mean_. The mean difference in PTC nodule E_max_ between positive and negative CLNM was 19.57 kPa, which was substantially larger than the 6.14 kPa difference observed for E_mean_ These results align with the percentage differences for both parameters, indicating a significantly larger disparity in E_max_ compared to E_mean_ (27.51% vs. 12.9%). These findings, coupled with a high odds ratio of 4.84 and the “strong effect” interpretation zone of SMD, suggest that E_max_ exhibits a more pronounced increase in positive CLNM patients compared to E_mean_. This pattern was consistently observed in our 2 × 2 contingency table analysis, where E_max_ significantly achieved a 0.07 higher AUC value. Although the sensitivity for E_max_ decreased by 6%, its specificity improved substantially by 18%. This indicates that the maximum elasticity indices could be practical in avoiding unnecessary surgical interventions. Possible clinical factors affecting E_mean_ include carotid pulse interference and nodule composition (such as the presence of a cystic component in some cases), which may lower the sensitivity and diagnostic values of E_mean_. Meanwhile, E_max_ is less influenced by these factors and offers more consistency, which may explain its superior performance compared to E_mean_ in our findings.

Recognizing central lymph node involvement is crucial, as it significantly impacts overall survival rates and the extent of surgical procedures [31]. For instance, the American Thyroid Association Guidelines (ATA) suggest that lobectomy is adequate for unifocal, intrathyroidal PTC less than 1 cm without a prior history of radiation and central lymph node metastasis [32]. Therefore, a positive CLNM will necessitate a change in the approach to a more extensive lymph node dissection procedure. Conventional ultrasound is the first and most commonly used modality for preoperative assessment of lymph node metastasis. However, its low sensitivity (37–84%) and operator dependency hinder its reliability [33]. USE has demonstrated remarkable effectiveness in other fields, such as liver fibrosis determination and hepatocellular carcinoma differentiation [34]. Its unique ability to quantify tissue stiffness allows for stricter differentiation between lesions and reduces operator dependency, making it more sensitive and specific, thus enhancing its clinical appeal. A systematic review conducted by Yan Xue et al. evaluated the effectiveness of SWE in diagnosing breast lesions, yielding a sensitivity and specificity range from 84 to 92% across diverse ethnicities, with an ICC ratio of 0.8, promising high reliability and reproducibility [35, 36]. We established good AUC values for E_max_ and E_mean_ in differentiating positive and negative CLNM, confirming the high USE value in cervical lymph node assessment.

Recent advancements in multimodal ultrasound approaches, including ARFI imaging, provide valuable insights into tissue characteristics. These methods show potential to complement SWE by addressing factors such as vascularity and stiffness disparity [37]. Our analysis revealed high statistical heterogeneity in the pooled mean difference of elastography parameters between positive and negative CLNMs. This variability is likely due to differences in patient characteristics across participating centers and inconsistencies in the protocols used for USE examinations. We conducted a subgroup analysis among the reviewed elastography methods to address this issue. SWE and ARFI, as the two primary USE techniques, demonstrated comparable effectiveness in distinguishing between CLNMs. However, ARFI shows more consistent results with lower heterogeneity across studies. This suggests that subgrouping based on USE techniques reduces the heterogeneity, and ARFI may offer greater reliability and generalizability than SWE. Lastly, while studies had conflicting findings about the potential influence of age on elastographic parameters, our meta-regression analysis showed that no such association exists [38]. The same was true for thyroid nodule size. Similarly, we analyzed the effect of thyroid nodule size on elastographic outcomes. Contrary to some literature claiming the impact of nodule size on elastographic indices, our findings revealed no significant correlation between elastographic parameters and size variables [39]. Consistent with findings from other studies, including Jung et al., our results demonstrate that combining SWE with conventional ultrasound enhances differentiation performance. This combination improves the AUC by 0.09 (0.85 for the combination vs. 0.76 for Emax) and sensitivity by 0.22 (0.80 for the combination vs. 0.58 for Emax). Interestingly, specificity remains unchanged compared to E_max_ [40]. By switching to USE mode with ultrasound during cervical ultrasonography examinations and integrating these two modalities, the most accurate results in assessing the status of CLNM are achieved. In our study, the integration of USE with conventional ultrasound yielded an AUC of 0.85, sensitivity of 80%, and specificity of 79% for CLNM detection. This performance substantially exceeds conventional ultrasound benchmarks reported in a recent meta-analysis [41] which documented only 28% sensitivity for central compartment metastasis despite 95% specificity. Our combined approach addresses this critical sensitivity gap, demonstrating a 52% absolute improvement in metastasis detection capability for anatomically challenging regions. The high AUC further confirms that elastography integration optimizes the risk-benefit balance, reducing false negatives compared to conventional ultrasound alone while maintaining clinically actionable specificity. These findings validate USE as a force multiplier for conventional ultrasound, particularly where its standalone performance is suboptimal. This combined approach leverages the strengths of both imaging techniques and highlights their complementary roles in enhancing classification performance.

While USE demonstrated a clear advantage in our study, several limitations must be acknowledged to contextualize the findings and guide future research: (1) A large proportion of the studies included in this analysis were conducted in East Asian countries. This geographical concentration may limit the generalizability of the findings to populations in Western countries or other regions with distinct demographic, genetic, and clinical profiles. Further validation of USE in diverse populations is essential to establish its universal applicability. (2) The current study primarily focused on two elastographic parameters, E_mean_ and E_max_, while other potentially valuable parameters were either not assessed or scarcely evaluated in the included studies. These alternative parameters might offer additional diagnostic insights or complement Emean and Emax in clinical practice. Future research should explore these parameters systematically to determine their clinical relevance and comparative utility. (3) This study concentrated on the role of USE in differentiating positive and negative CLNMs. However, USE’s diagnostic performance could be enhanced by integrating it with other clinical variables, such as serum biomarkers, tumor characteristics, or advanced imaging modalities. Investigating the combined diagnostic value of USE with these variables represents a promising direction for future studies. (4) strain elastography (SE) studies were excluded from quantitative synthesis due to methodological incompatibility and limited evidence. SE relies on tissue deformation under manual compression, producing semi-quantitative outputs (e.g., elasticity scores) fundamentally distinct from the acoustic radiation force principles underlying SWE/ARFI. This exclusion preserves analytical homogeneity but highlights a literature gap: future studies should develop SE-specific quantitative frameworks for CLNM assessment. (5) A notable limitation of the present meta-analysis is the predominant focus on central (level VI) lymph node metastases, with most studies either excluding lateral (levels II–V) nodes or reporting only combined cervical outcomes. This absence of compartment-specific data precluded formal subgroup meta‐analysis by nodal level. These observations underscore the potential for improved discrimination of lateral metastases when node-specific thresholds are applied. We therefore recommend that future diagnostic accuracy studies report elastographic parameters separately for central and lateral nodal compartments.

## Conclusion

In summary, USE, as a simple and non-invasive method, demonstrates significant potential in improving the discrimination accuracy for central lymph node metastasis in PTC examination. Among the two parameters analyzed, Emax notably outperforms Emean in terms of differentiation values, providing a reliable tool to differentiate positive from negative CLNM, thus helping to avoid unnecessary surgeries. Moreover, the integration of USE with conventional ultrasound enhances preoperative evaluations, offering more precise assessments before surgery.

## Supplementary Information

Below is the link to the electronic supplementary material.

Supplementary Material 1

Supplementary Material 2

Supplementary Material 3

Supplementary Material 4

## Data Availability

The data supporting this article can be obtained from the corresponding author upon request.

## Declarations

### Animal study

N/A.

## Abbreviations

USE: Ultrasound elastography
PTC: Papillary thyroid carcinoma
CLNM: Cervical lymph node metastasis
ARFI: Acoustic radiation force impulse
SWE: Shear wave elastography
RTE: Real-time elastography
AUC: Area under curve
DOR: Diagnostic odds ratio
ROC: Receiver operating characteristic
