# Supplementary material for: Determination of lymph node metastasis using quantitative ultrasound elastography of papillary thyroid carcinoma nodule: a systematic review and meta-analysis
Source: BMC Med Imaging. 2025 Aug 21;25:342. doi: 10.1186/s12880-025-01858-z (PMC12369039; doi:10.1186/s12880-025-01858-z)
Supplement: Supplementary file 1 — Supplementary Material 1 [file 12880_2025_1858_MOESM1_ESM.docx]

| Searched database | Search syntax |
| --- | --- |
| PubMed, Cochrane library, Web of Science and Embase | (elastography OR elastogram OR acoustography OR "shear" OR "sonoelastography" OR "elasticity" OR "stiffness" OR "Acoustic Radiation Force Impulse" OR Sonoelastography OR "shear wave" OR SWV OR kPa OR "velocity") AND papillary AND thyroid AND lymp* |

All databases were searched through Ovid simultaneously.
