# Supplementary figures and images for: Determination of lymph node metastasis using quantitative ultrasound elastography of papillary thyroid carcinoma nodule: a systematic review and meta-analysis
Source: BMC Med Imaging. 2025 Aug 21;25:342. doi: 10.1186/s12880-025-01858-z (PMC12369039; doi:10.1186/s12880-025-01858-z)

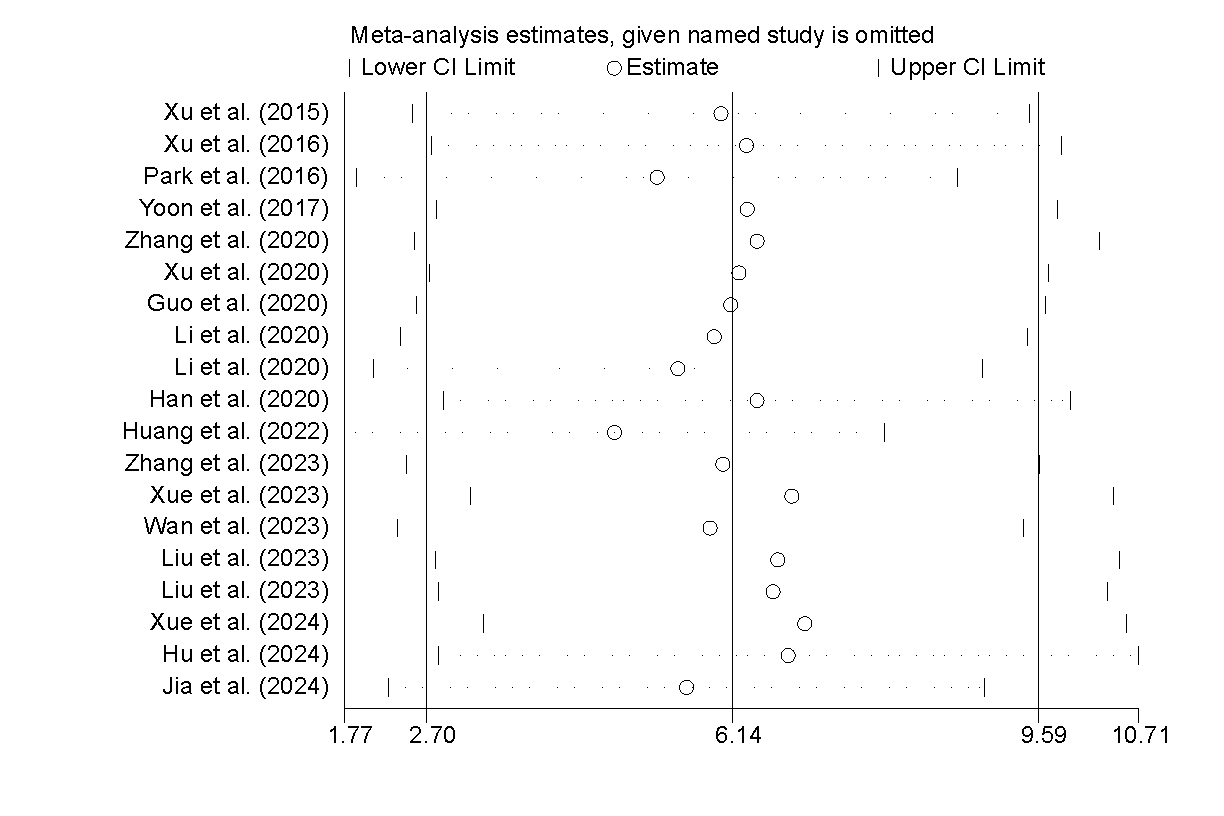

Supplement: Supplementary file 2 — Supplementary Material 2 [file 12880_2025_1858_MOESM2_ESM.docx]
