# Supplementary material for: Determination of lymph node metastasis using quantitative ultrasound elastography of papillary thyroid carcinoma nodule: a systematic review and meta-analysis
Source: BMC Med Imaging. 2025 Aug 21;25:342. doi: 10.1186/s12880-025-01858-z (PMC12369039; doi:10.1186/s12880-025-01858-z)
Supplement: Supplementary file 3 — Supplementary Material 3 [file 12880_2025_1858_MOESM3_ESM.docx]

**Differentiation performance of ultrasound elastography for cervical lymph node metastasis in patients with papillary thyroid carcinoma nodules: A systematic review and meta-analysis.**

**1. Review question:**

To determine the differentiation performance of ultrasound elastography for cervical lymph node metastasis (LNM) in patients with papillary thyroid carcinoma (PTC) nodules

**2. Searches:**

A detailed, comprehensive search will be conducted by two reviewers (S. Mohammadzadeh and A. Mohebbi) independently using online resources. Literature from PubMed, Web of Science, Embase, and Cochrane Library database will be included in the review. Observational studies (cross-sectional, case-control, cohort) and experimental articles (RCT, non-randomized trials) will be investigated for eligibility. Studies without desired data, reviews, meta-analyses, editorials, and commentaries will be excluded. Language limitations will not be applied.

The reviewers will use a multi-step, concise process to ensure that all the relevant, high-quality papers are included in the present study. Initially, they will independently review titles and abstracts to evaluate suitability. Then, duplicate studies will be removed, and the remaining articles will undergo a full-text assessment. We will follow Preferred Reporting Items for a Systematic Review and Meta-analysis of Diagnostic Test Accuracy Studies (PRISMA-DTA) guidelines for transparency and reproducibility.

(elastography OR elastogram OR acoustography OR "shear" OR "sonoelastography" OR "elasticity" OR "stiffness" OR "Acoustic Radiation Force Impulse" OR Sonoelastography OR "shear wave" OR SWV OR kPa OR "velocity") AND papillary AND thyroid AND lymp*

**3. Types of study to be included:**

Observational studies (cross-sectional, case-control, cohort) and experimental articles (RCT, non-randomized trials) will be included in our study.

**4. Condition or domain being studied:**

LNM is a critical factor in the staging, treatment planning, and prognosis of PTC. Accurate preoperative identification of metastatic lymph nodes is crucial for determining the extent of surgical intervention and postoperative management. While conventional ultrasound remains the primary imaging modality for evaluating cervical lymph nodes, its accuracy in differentiating benign from malignant nodes is limited. Ultrasound elastography (USE) has emerged as a promising non-invasive technique for assessing tissue stiffness, potentially improving the diagnostic accuracy of cervical lymph node evaluation. USE is based on the principle that malignant tissues tend to be stiffer than benign ones, allowing for potential differentiation between metastatic and reactive lymph nodes.

Several elastography techniques have been developed, including strain elastography and shear wave-based elastography, each with its methodologies for quantifying tissue elasticity. While preliminary studies have shown promising results in applying ultrasound elastography for cervical lymph node assessment, its role in clinical practice, particularly for patients with PTC, remains to be fully established.

This systematic review and meta-analysis aims to comprehensively evaluate the diagnostic performance of ultrasound elastography in differentiating cervical lymph node metastasis in patients with PTC. By synthesizing the available evidence, we seek to determine the overall accuracy, sensitivity, and specificity of this technique, as well as to identify potential factors influencing its diagnostic efficacy. The findings of this study may provide valuable insights for radiologists and clinicians in optimizing the preoperative assessment of cervical lymph nodes in PTC patients, potentially improving patient management and outcomes.

**5. Participants/population:**

Studies assessing quantitative USE parameters (e.g., Emean, Emax) for histopathologically confirmed PTC nodules will be included. The histopathological findings of lymph nodes must have been the base as the gold standard.

**6. Intervention(s), exposure(s):**

USE on pathologically confirmed PTC nodules.

**7. Comparator(s)/control:**

Pathology will be used as the ground truth, and the diagnostic performance of USE will be compared to pathology.

**8. Main outcome:**

The main aim is to demonstrate the classification performance of shear wave elastography using sensitivity, specificity, and diagnostic odds ratio (DOR) for Emean and Emax.

**9. Additional outcome(s):**

A) The summary receiver operator curve (ROC) will be plotted, and Area Under the Curve (AUC) will be presented to enhance predictive assessment.

B) Combined role of USE to conventional ultrasound will be assessed using sensitivity, specificity, AUC, etc.

C) To calculate reactive and metastatic lymph node Emean and their mean difference (MD), standardized mean difference (SMD) and percentage difference, if enough data are reported.

D) To calculate reactive and metastatic lymph node Emax and their MD, SMD, and percentage difference, if enough data are reported.

E) To evaluate publication bias on upper results if enough studies are included.

F) To perform sensitivity analysis on upper analyses if enough studies are included.

G) To investigate the possible sources of statistical heterogeneity and subgroup based on QUADAS-2, type of USE scanner type, size, study design, age, etc., if enough studies are included for making this

H) To perform GRADE assessment on upper results.

**10. Data extraction (selection and coding):**

After a brief title and abstract screening for relevance, two reviewers (A. Mohebbi and S. Mohammadzadeh) will independently read the full-text articles. The methods and materials section will be scrutinized by both reviews for methodological robustness to ensure reliable and reproducible results. As the next step, results will be evaluated, and outcome parameters will be extracted. The extracted data will be entered into an Excel spreadsheet, including details such as study characteristics (author, year, study design), patient demographics, nodule characteristics, elastography parameters, and diagnostic performance metrics (sensitivity, specificity, AUC, etc.).

Disagreements (methodological accountability disagreement) will be resolved through discussion with a third reviewer. Once all data are compiled, statistical and summary measures will be calculated.

**11. Risk of bias assessment:**

Quality Assessment of Diagnostic Accuracy Studies-2 (QUADAS-2), will be used to assess bias

of included studies, along with some additional comments recommended by the Cochrane

Handbook for Systematic Reviews of Diagnostic Test Accuracy.

**12. Strategy for data synthesis:**

The analysis will be conducted using STATA version 17.0. As recommended by the Cochrane Handbook for Systematic Reviews of Diagnostic Tests Accuracy for diagnostic studies, a random-effects model will be used. The I2 ≥ 50% will be considered a high statistical heterogeneity

model.

**13. Contact details for further information:**

Afshin Mohammadi

mohammadi.a@umsu.ac.ir

**14. Organizational affiliation of the review:**

Students’ Scientific Research Center (SSRC), Tehran, Iran.

**15. Review team members and their organizational affiliations:**

Alisa Mohebbi

Saeed Mohammadzadeh

Mohammad Ghaffari

**16. Type and method of review:**

Systematic review and meta-analysis

**17. Anticipated or actual start date:**

March 10, 2025

**18. Anticipated completion date:**

May 10, 2025

**19. Funding sources/sponsors:**

None

**20. Conflicts of interests:**

None
