# Supplementary material for: Determination of lymph node metastasis using quantitative ultrasound elastography of papillary thyroid carcinoma nodule: a systematic review and meta-analysis
Source: BMC Med Imaging. 2025 Aug 21;25:342. doi: 10.1186/s12880-025-01858-z (PMC12369039; doi:10.1186/s12880-025-01858-z)

Appendix D: Sensitivity and specificity for ultrasound elastography added to conventional ultrasound in differentiating positive and negative CLNMs.


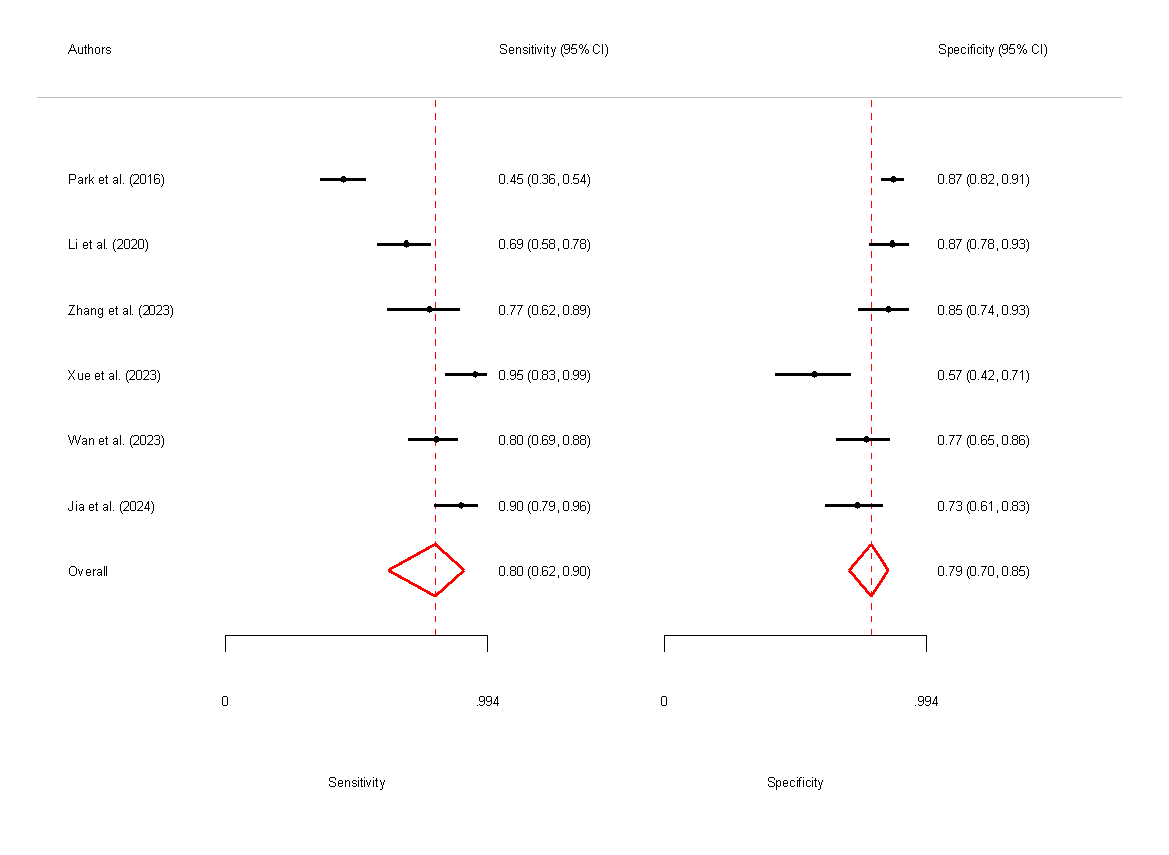


ROC curve of combined ultrasound and elastography models in differentiating positive and negative CLNMs.


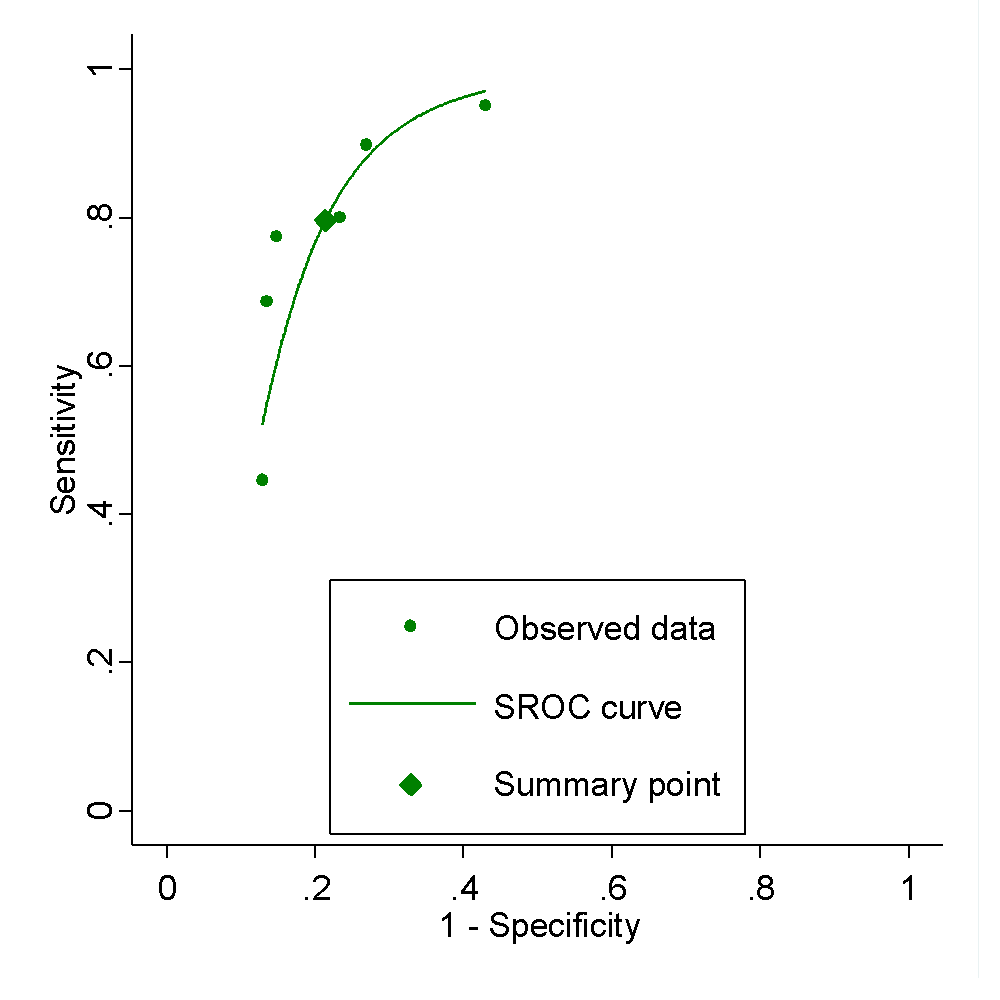

Supplement: Supplementary file 4 — Supplementary Material 4 [file 12880_2025_1858_MOESM4_ESM.docx]
